# CHANGING PARADIGMS IN PREOPERATIVE FASTING: RESULTS OF A JOINT EFFORT IN PEDIATRIC SURGERY

**DOI:** 10.1590/0102-6720201700010003

**Published:** 2017

**Authors:** Carlos Augusto Leite de Barros CARVALHO, Augusto Aurélio de CARVALHO, Paulo Luiz Batista NOGUEIRA, José Eduardo de AGUILAR-NASCIMENTO

**Affiliations:** 1 Santa Casa de Misericórdia de Cuiabá Hospital; 2 Faculty of Medicine, University Center of Varzea Grande, Várzea Grande, MT, Brazil.

**Keywords:** Fasting, Ambulatory surgical procedures, Pediatrics

## Abstract

**Background:**

Current researches associate long fasting periods to several adverse consequences. The fasting abbreviation to 2 h to clear liquids associated with the use of drinks containing carbohydrates attenuates endocrinometabolic response to surgical trauma, but often is observed children advised to not intake food from 00:00 h till the scheduled surgical time, regardless of what it is.

**Aim:**

To evaluate the safety of a protocol of preoperative fasting abbreviation with a beverage containing carbohydrates, and early postoperative feeding in children underwent elective small/mid-size surgical procedures during a national task-force on pediatric surgery.

**Methods:**

Thirty-six patients were prospectively included, and for several reasons five were excluded. All 31 who remained in the study received a nutritional supplement containing 150 ml of water plus 12.5% maltodextrin 2 h before the procedure. Data of the pre-operative fasting time, anesthetic complications and time of postoperative refeeding, were collected.

**Results:**

Twenty-three (74.2%) were males, the median age was 5 y, and the median weight was 20 kg. The median time of pre-operative fasting was 145 min and the time of post-operative refeeding was 135 min. There were no adverse effects on the anesthetic procedures or during surgery. Post-operatively, two children (6.5%) vomited.

**Conclusion:**

The abbreviation of pre-operative fasting to 2 h with beverage containing carbohydrate in pediatric surgery is safe. Early refeeding in elective small/mid-size procedures can be prescribed.

## RESUMO

**Racional:**

Trabalhos atuais associam longos períodos de jejum com diversas consequências adversas. A abreviação do jejum de 2 h para líquidos claros associado ao uso de bebidas contendo carboidratos atenua resposta endocrinometabólica ao trauma, porém frequentemente as crianças são orientadas a não ingerir alimentos a partir das 00:00 h do dia anterior à operação, independente do horário do procedimento cirúrgico.

**Objetivo:**

Avaliar a segurança de um protocolo de abreviação do jejum pré-operatório, com o uso de bebida contendo carboidratos, e realimentação precoce.

**Métodos:**

Foram avaliados prospectivamente 36 crianças submetidas a procedimentos cirúrgicos eletivos de pequeno e médio porte. Cinco foram posteriormente excluídos do estudo. Todos os 31 remanescentes receberam suplemento nutricional com maltodextrina 12,5% em 150 ml de água aproximadamente 2 h antes do procedimento. Foram coletados dados do tempo de jejum pré-operatório, complicações anestésicas e tempo de realimentação.

**Resultados:**

Vinte e três (74,2%) eram do gênero masculino, com idade mediana de cinco anos e peso mediano de 20 kg. O tempo mediano de jejum pré-operatório foi de 145 min e o tempo mediano para realimentação foi de 135 min. Não houve eventos adversos durante a anestesia ou operação. No período pós-operatório, duas (6,5%) crianças vomitaram.

**Conclusão:**

A abreviação de jejum pré-operatório para 2 h com uso de bebida contendo carboidratos, em operações eletivas de crianças, é seguro e não está associado ao maior risco de broncoaspiração pulmonar. Realimentação precoce pode ser prescrita nos procedimentos cirúrgicos analisados.

## INTRODUCTION

Recent studies have changing the paradigm of perioperative feeding in children. Currently, the abbreviation of preoperative fasting and early feedback points are very important in the recovery of operated children.

Although security is well established in preoperative abbreviation of fasting time^13^ ^,^ ^18^ ^,^ ^24^, we observe high strength from conventional services in pediatric surgery to implement this approach^7^. Likewise, studies show that early refeeding after operation is safe and optimizes postoperative recovery^10^.

Preoperative fasting was widespread from 1946 when Mendelson noted a small number of cases of bronchopulmonary aspiration in pregnant women undergoing general anesthesia^20^. A study at Children's Hospital Royal Aberdeen in the UK showed that the average period of preoperative fasting time to clear and solid liquid was 8-12 h^12^. However, in the literature there are studies showing that the gastric emptying time in healthy children is relatively short and prolonged fasting exacerbates the metabolic status of patients. There is a greater concentration of ketone bodies in the blood plasma of children who spent more than 4 h of fasting compared to those with only 2 h, indicating that fasting increases catabolism, increasing surgical stress^4^.

In infants and children, the preoperative fasting can be minimized with the provision of clear liquids up to 2 h before the procedure. This approach is already accepted worldwide for various societies of anesthesiology^3^ ^,^ ^21^ ^,^ ^27^. The prescription of these liquids helps preserve intravascular volume, improving hemodynamics and facilitating obtaining peripheral venous access. Shorten the preoperative fasting contributes to reducing hunger, thirst and anxiety of patients, and so they show greater cooperation at the time of anesthesia^25^. Furthermore, the intake of drinks enriched with carbohydrates reduces the organic response to the surgical trauma^1^.

Likewise, the reintroduction of the diet should be stimulated early, not observing changes in the incidence of food intolerance, nausea or vomiting. In Brazil, these recommendations have been implemented by the ACERTO (Accelerating Total Recovery Postoperative) protocol with obvious results in improving the recovery of patients in services that had it implanted^2^. The Brazilian Association of Pediatric Surgery recently recommended the ACERTO Protocol in the country^5^.

There are no national studies on the use of preoperative fasting abbreviation protocols in children, and thus the aim of this pilot study was to evaluate the safety of a short protocol of preoperative fasting, using drink carbohydrates, and also early refeeding in children undergoing small and medium-sized elective surgeries.

## METHOD

Were included in a prospective way, children candidates for elective surgeries at the Santa Casa de Misericórdia de Cuiabá Hospital, Cuiabá, MT, Brazil, on May 7, 2016, during a campaign for pediatric operations marked nationally (X Joint Effort for Child Surgery ). All parents and guardians were informed about the study and signed a consent form and cleared up after approval of the Ethics Committee in Research under number 51932515400005541. Following the guidelines of the ACERTO protocol, all patients remained fasting for solid for at least 6 h received drink containing carbohydrate (maltodextrin 12.5%, 150 ml) over 2 h before starting the operation. 

Upon entrance of the patient to the operating room, was asked to escort people if the child had ingested the drink containing carbohydrate; the intake time; and if he/she had hunger. These data were confirmed with the medical records. Were also collected data on age, weight, diagnosis, comorbidities, start time of anesthesia, the end of the operation time, if there were complications during the procedure, postoperative diet start time, presence of postoperative symptoms and discharge time.

Hospital discharge was determined according to the following parameters: fully awake child, hemodynamically stable, without vomiting and pain controlled. In patients who were discharged without postoperative feeding, was recommended to start light diet when get home. When this occurred, was asked to inform when the refeeding was carried out. Parents and/or guardians were instructed to return with the child in outpatient consultation between 7-14 days for postoperative follow-up.

### Statistical analysis

The data were analyzed using the Statistical Package for Social Sciences (SPSS) Version 22. Continuous data were presented as median and interquartile range (IQR).

**TABLE 1:** Demographic and clinical data of X Joint Effort for Child Surgery at Santa Casa Hospital of Cuiabá, MT, Brazil (n=31)

	n	%
Gender		
Male	23	74.2
Female	8	25.8
Age (years)*	5 (2-8)	
Weight (kg)*	20 (14-32)	
Diagnosis		
Postectomy	12	38.7
Dermoid cyst extraction	7	22.6
Umbilical hernia	4	12.9
Unilateral inguinal hernia	4	12.9
Bilateral inguinal hernia	2	6.5
Unilateral orquidopexy	2	6.5

* median and interquartile range

**FIGURE 1:**
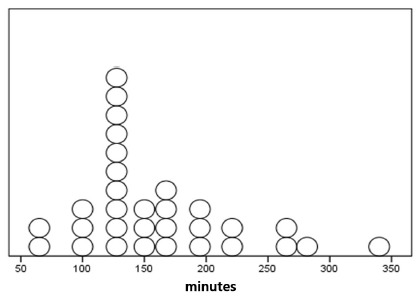
Fasting time distribution preoperatively in minutes (n=31)

Note: Each circle represents the preoperative fasting period of each patient

**FIGURE 2:**
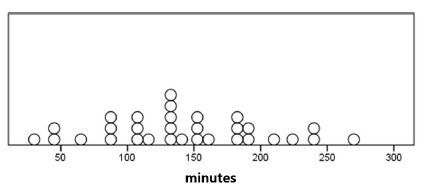
Postoperative refeeding time distribution in minutes (n=31)

Note: Each circle represents the time after the operation in which each patient was refed

## RESULTS

Were initially included 36 patients, five were subsequently excluded for different reasons, namely: two for symptoms of infection of the upper airways and three for three different reasons (lack of postoperative follow-up, not having ingested the carbohydrate drink before the procedure, and parents did not allow the participation in the study).

The main reason for the operation was phimosis (38.7%), followed by dermoid cyst (22.6%), inguinal hernia (19.4%), umbilical hernia (12.9%) and cryptorchidism (6.5%). Twenty-three (74.2%) patients were male with a median age of five years (IQR: 2-8 years) and median weight of 20 kg (IQR: 14-32 kg). None were associated with comorbidities.

The median time of preoperative fasting was 145 min (IQR: 120-190 min). Parents/guardians reported that 64.5% (n=20) of the children showed signs of being hungry before surgery. There were no adverse events related to anesthesia or complications during surgery.

The median time for refeeding was 135 min (IQR: 105-185 min). In the postoperative period, two (6.5%) children vomited: one with nausea followed by an episode of vomiting during hospitalization and other three episodes after discharge during transport to their municipality.

## DISCUSSION

The American Society of Anesthesiologists recommends for patients of any age, previously healthy and who will undergo elective surgical procedures with the use of anesthesia, fasting periods of 2 h to clear liquids (water, juices without pulp, carbohydrate drinks, teas); 4 h breast milk; 6 h for formula milk, non-human milk and snacks (toast and clear liquids); and 8 h to fatty foods or fried food^3^.

However, pediatric patients often remain for a prolonged period of preoperative fasting, due to normal sleep period before the procedure^22^. These long periods of food abstention can lead to thirst and dehydration, hunger, hypoglycemia (especially children who have lower reserves of glycogen), irritability, headache and delay in coming up from anesthesia, causing unpleasant experience for these small patients^9^ ^,^ ^15^. In addition, 13.5% of children presenting for elective surgery are not suitable with fasting, and it is observed that most parents do not know the real cause of the need for food deprivation preoperatively - only 9% believe is due to risk of aspiration^8^. Failure to comply with the preoperative fasting guidance often results in delays and operation cancellations^17^.

Children using liquid until 2 h before anesthesia preoperative period have less hunger and thirst, lower rates of dehydration, and better hemodynamic stability compared to habitual fasting^7^ ^,^ ^23^ ^,^ ^28^. A systematic review of the Cochrane Database showed that children with liquids up to 120 min before surgery, besides presenting with less thirst and hunger, best behaved and comfortable than those who remained in regular fasting^6^.

Gastric emptying for liquids in children is fast. In a study was offered volume of 7 ml/kg to 16 volunteers aged 6-14 years and was observed by MRI that the average gastric emptying time was only 30 min^26^. Schmidt et al.^25^ found no difference in residual volume or gastric pH in children after 1-2 h clear fluid intake^9^. Pulmonary aspiration is rare event with the modern techniques of anesthesia. Anderson et al. ^4^ found an incidence of 0.03% of pulmonary aspiration in more than 10,000 elective surgical procedures in pediatrics.

Studies in adults found that the use of drinks containing carbohydrates in the preoperative period determines greater satisfaction, less irritability, lower incidence of vomiting, better gastric emptying and especially smaller organic response to surgical stress^16^. In children it has also been observed that the use of carbohydrate drinks before the surgical procedure is safe and is associated with lower insulin resistance indexes in the postoperative period^14^.

This pilot study refers to a number of cases of children operated electively in which has been used a fasting abbreviation protocol with maltodextrin 12.5% ​​in 150 ml of water 2 h before operation. It was observed that the drink containing carbohydrate had good acceptance in the pediatric population and there were no anesthetic complications that could be associated with reduced preoperative fasting time, as vomiting in anesthesia, pulmonary aspiration, laryngospasm or greater use of anesthetics during the procedure. Furthermore, the incidence of postoperative vomiting was 6.5% below what has been reported in the literature (range 9-70%)^11^ ^-^ ^19^. There is a need for further comparative studies determining other clinical and metabolic benefits of the abbreviation of preoperative fasting in elective surgeries, such as less surgical stress response, better glycemic stability, postoperative lower insulin resistance and patient faster recovery. In this context, it should be applauded the recent position of the Brazilian Pediatrics Surgery Association^5^ in adopting new perioperative care protocols that promote the reduction of fasting before and after surgery.

## CONCLUSION

The abbreviation of preoperative fasting with the use of clear liquids (carbohydrate beverage) in elective operations of children is feasible, safe and is not associated with increased risk of pulmonary aspiration. Early refeeding in small and medium-sized pediatric procedures is feasible and safe.
